# Water Influx through the Wetted Surface of a Sweet Cherry Fruit: Evidence for an Associated Solute Efflux

**DOI:** 10.3390/plants9040440

**Published:** 2020-04-02

**Authors:** Andreas Winkler, Deborah Riedel, Daniel Alexandre Neuwald, Moritz Knoche

**Affiliations:** 1 Institute of Horticultural Production Systems, Leibniz University Hannover, Herrenhäuser Straße 2, 30419 Hannover, Germany; andreas.winkler@obst.uni-hannover.de (A.W.); deborah.riedel@gmx.de (D.R.); 2 Competence Centre for Fruit Growing—Lake Constance (KOB), Schumacherhof 6, 88213 Ravensburg, Germany; Neuwald@kob-bavendorf.de

**Keywords:** *Prunus avium*, cracking, leakage, osmolyte, uptake, penetration

## Abstract

Sweet cherries are susceptible to rain-cracking. The fruit skin is permeable to water, but also to solutes. The objectives of this study were to (1) establish whether a solute efflux occurs when a sweet cherry fruit is incubated in water; (2) identify the solutes involved; (3) identify the mechanism(s) of efflux; and (4) quantify any changes in solute efflux occurring during development and storage. Solute efflux was gravimetrically measured in wetted fruit as the increasing dry mass of the bathing solution, and anthocyanin efflux was measured spectrophotometrically. Solute and anthocyanin effluxes from a wetted fruit and water influx increased with time. All fluxes were higher for the cracked than for the non-cracked fruit. The effluxes of osmolytes and anthocyanins were positively correlated. Solute efflux depended on the stage of development and on the cultivar. In ‘Regina’, the solute efflux was lowest during stage II (25 days after full bloom (DAFB)), highest for mid-stage III (55 DAFB), and slightly lower at maturity (77 DAFB). In contrast with ‘Regina’, solute efflux in ‘Burlat’ increased continuously towards maturity, being 4.8-fold higher than in ‘Regina’. Results showed that solute efflux occurred from wetted fruit. The gravimetrically determined water uptake represents a net mass change—the result of an influx minus a solute efflux.

## 1. Introduction

Rain cracking of sweet cherry is a serious economic problem in many regions of the world, where this high-value crop is produced [1,2]. Water influx through the fruit skin (osmotic) is considered an important factor in rain cracking [3].

Transport across the epidermal cuticle represents the rate-limiting step in water influx in a fruit [4]. Therefore, in a detailed investigation of fruit water relations, particular attention should be given (1) to the cuticle’s permeability characteristics and (2) to the fruit’s water potential, especially to the components that make up this water potential.

The cuticle of the sweet cherry fruit is permeable to water and, to a lesser extent, to small polar molecules, such as glucose and fructose [5]. These two sugars are both major osmolytes in sweet cherry fruit [6]. Studies of the sweet cherry fruit’s water potential established that the water potential markedly decreases towards maturity. Furthermore, the turgor of a mature fruit is negligibly low, relative to the very negative osmotic potential of its juice [7,8]. Sweet cherry is not unique in this respect. Similar conclusions have been reached for grapes (*Vitis vinifera* L.) [9,10]. The low cell turgor in grape berries is thought to result from the high concentration of osmolytes in the cell-wall free space (apoplast). This almost eliminates any osmotic potential difference across the plasma membrane (i.e., between the apoplast and the symplast) and, thus, the cell turgor [9,10,11,12]. Whether the same situation also applies to the sweet cherry fruit, is not known. Nevertheless, based on the above, it would seem plausible that the osmotic potential of the apoplastic solution in a sweet cherry should be very close to that in the symplast.

Combining the above findings, it would seem fair to hypothesize that some of the key solutes occurring in the apoplast would diffuse out of the fruit, when it is incubated in deionized water. The rate of solute efflux would be expected to change during development and to vary among cultivars.

Growth of the sweet cherry fruit follows a double sigmoidal pattern that is referred to as stages I, II, and III [13,14]. During stage I, cell division occurs in the pericarp, during stage II, the pit and the embryo develop with little change in the fruit mass. Stage III (also referred to as the final swell) is characterized by the accumulation of carbohydrates and a rapid increase in fruit mass, due to cell enlargement in the mesocarp. Based on the above, it is expected that solute efflux would increase as the osmotic potential of the apoplast becomes more negative as the fruit matures during stage III.

The objectives of this study were (1) to establish whether a solute efflux occurs when a sweet cherry fruit is incubated in water; (2) and, if so, to identify the solutes involved and their efflux rates; also (3) to identify the mechanism(s) of such a solute efflux; and (4) to quantify any changes in solute efflux rate occurring preharvest, during fruit development and also postharvest, during fruit storage.

## 2. Results

**Figure 1:**
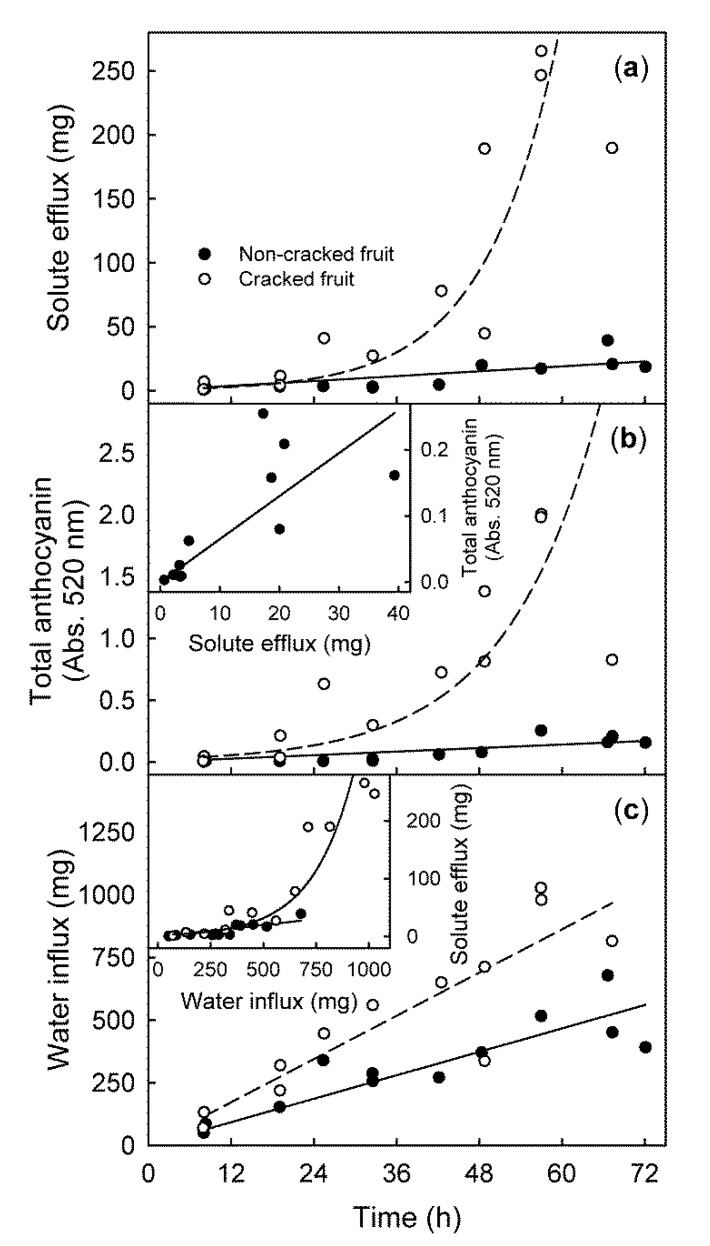
Time courses of the solute efflux (**a**), anthocyanin efflux (**b**), and water influx (**c**) from fruit that was incubated in deionized water. Data for the cracked and the non-cracked fruit were analyzed separately. Inset (**b**) Relationship between the anthocyanin efflux and the efflux of solutes. Inset (**c**) Relationship between the efflux of the solutes and the water influx. Anthocyanin content was determined by quantifying the absorption at 520 nm (Abs. 520 nm).

The efflux of all solutes measured increased with time and was several-fold higher in the cracked than in the non-cracked fruit (Figure 1a). The increases in the efflux of all solutes over time, followed an exponential function for the cracked fruit (*Efflux* (mg) = *e*^0.095∗*time* (h)^; r^2^ = 0.81 ***) but followed a linear function for the non-cracked fruit (*Efflux* (mg) = 0.318 ∗ *time* (h); r^2^ = 0.62 ***) (Figure 1a). The efflux of solutes determined gravimetrically was linearly related to the soluble solids content of the residue, after being taken up in 2.5 mL of deionized water and measured by refractometry (Figure S1). Across all experiments, the regression equation was (*Efflux* (mg) = 26.88 ∗ *soluble solids content* (%); r^2^ = 1.00 ***, *n* = 266) (Figure S1). This relationship was valid regardless of the integrity of the fruit (i.e., cracked or non-cracked). The efflux of anthocyanins over time showed the same relationship as the efflux of all solutes (Figure 1b). The increases in efflux of anthocyanins over time, followed an exponential function for the cracked fruit (*Total anthocyanin* (Abs.at 520 nm) = 0.0233 ∗ *e*^0.0736∗*time* (*h*)^; r^2^ = 0.75 ***) but followed a linear function for the non-cracked fruit (*Total anthocyanin* (Abs.at 520 nm) = 0.0034 ∗ *time* (h) − 0.0534; r^2^ = 0.74 ***). Further, the efflux of anthocyanins was positively related to the solute efflux (r = 0.97 **) (Figure 1b Inset). Water influx increased linearly with time (Figure 1c). The rate of water influx was significantly higher for the cracked than for the non-cracked fruit (14.4 vs. 7.8 mg h^−1^) (Figure 1c). There was a linear relationship between the solute efflux and the water influx for the non-cracked fruit (*Efflux* (mg) = 0.040 ∗ *water influx* (mg), r^2^ = 0.83 ***), and an exponential relationship was observed between them for the cracked fruit (*Efflux* (mg) = *e*^0.0064∗*water influx* (mg)^, r^2^ = 0.79 ***) (Figure 1c Inset).

**Figure 2:**
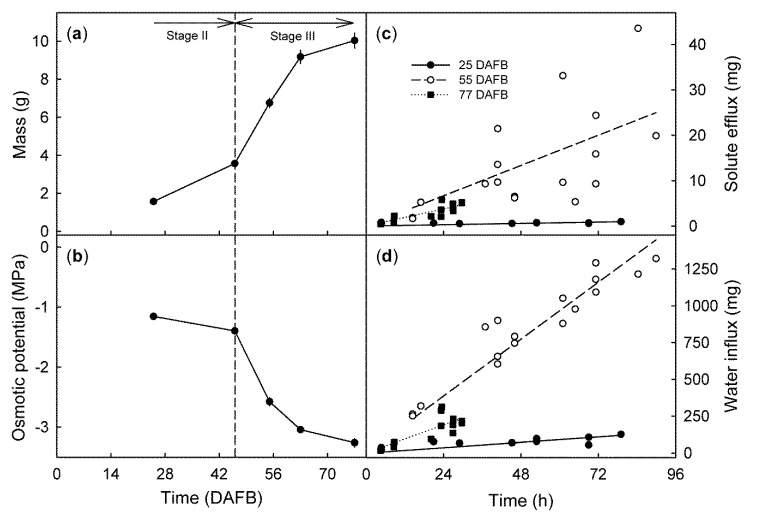
(**a**) Developmental time course of changes in fresh fruit mass and (**b**) osmotic potential of ‘Regina’ sweet cherries. Time courses of solute efflux (**c**) and of water influx (**d**) in developing the Regina fruit at 25, 55, and 77 days after full bloom (DAFB). At 77 DAFB, all fruit had cracked within 30 h of incubation. Horizontal arrows in (**a**) indicate stages II and III of fruit development, the vertical dashed line in (**a**,**b**) represents the stage II/III transition.

**Figure 3:**
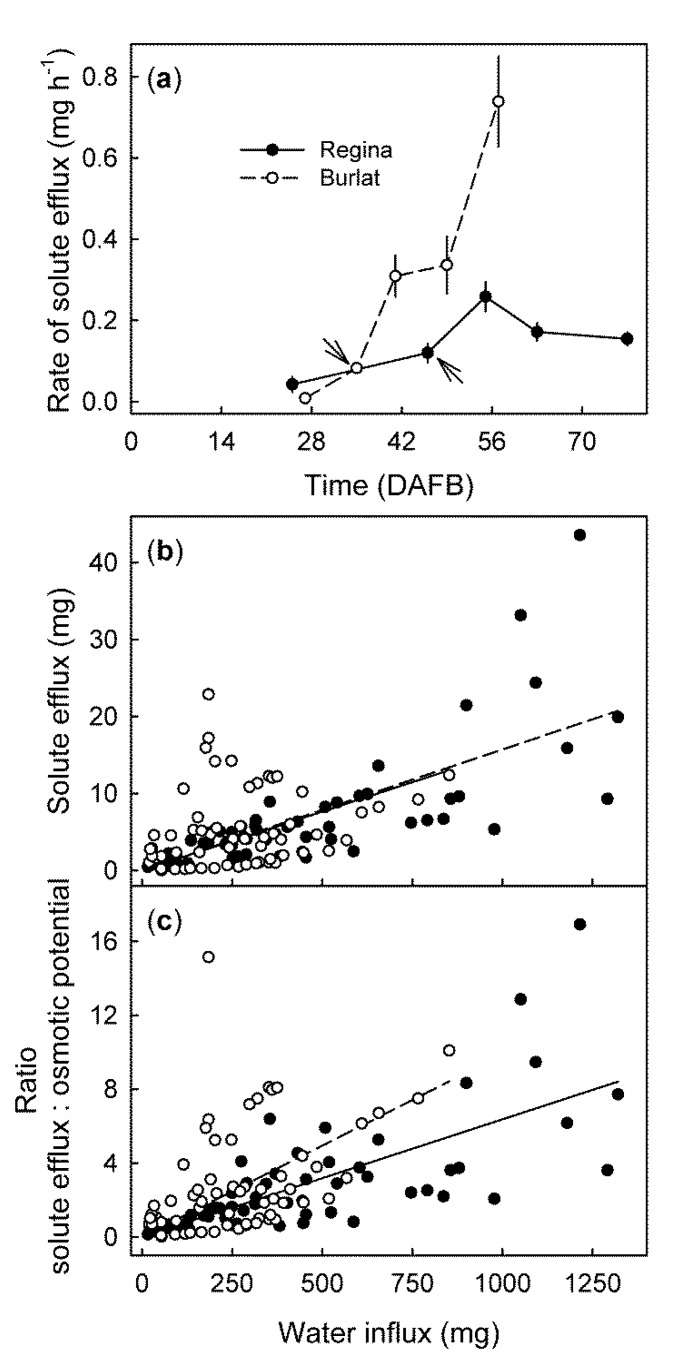
(**a**) Developmental time course of the rate of solute efflux from the ‘Regina’ and ‘Burlat’ sweet cherry fruit. Relationship between the solute efflux and the water influx (**b**), and the ratio of solute efflux divided by the negative osmotic potential and water influx (**c**). DAFB = days after full bloom. The arrows in (**a**) represent the stage II/III transitions in ‘Regina’ and ‘Burlat’.

From stage II to maturity, fruit mass increased (Figure 2a) and osmotic potential decreased (Figure 2b) with time, in a sigmoid manner. The time courses of solute efflux (Figure 2c) and water influx (Figure 2d) were linear for all developmental stages, the rates depended on the stage of development. In ‘Regina’ they were lowest for stage II fruit (25 DAFB), highest for mid stage III fruit (55 DAFB), and then decreased slightly for the mature fruit (77 DAFB) (Figure 3a). Solute efflux from ‘Burlat’ was markedly higher than from ‘Regina’. Solute efflux increased continuously during development (Figure 3a). For both cultivars, a common, somewhat-variable but consistent positive relationship was obtained between the rates of solute efflux and of water influx (Figure 3b). For both cultivars, the ratio of the solute efflux rate divided by the negative of the juice osmotic potential was considered to be an efflux rate normalized for differences in the osmolyte concentration of the juice. Interestingly, following normalization of the solute effluxes per unit of water influx, the values were slightly higher for ‘Burlat’ than for ‘Regina’ (Figure 3c).

**Figure 4:**
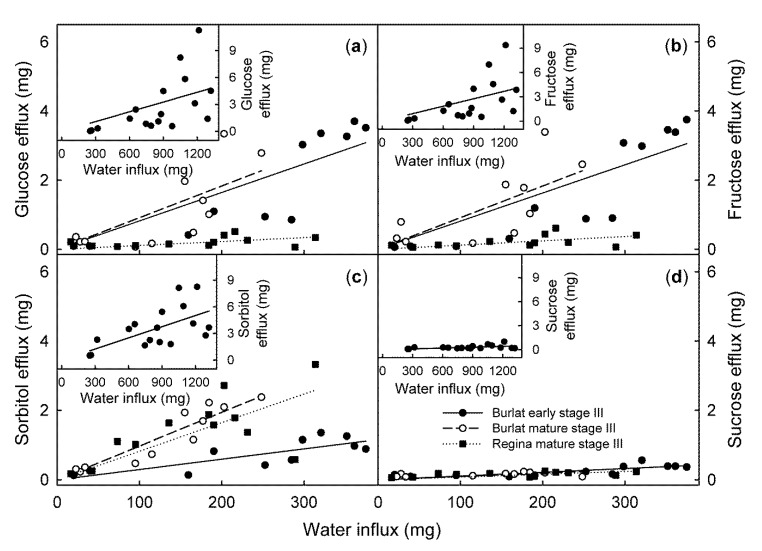
Efflux of glucose (**a**), fructose (**b**), sorbitol (**c**), and sucrose (**d**), as affected by the water influx of early stage III and mature stage III ‘Burlat’ and ‘Regina’ sweet cherry fruit. Due to the higher efflux, the early stage III ‘Regina’ fruit is plotted on a different scale in the insets. The mature stage III ‘Regina’ is drawn in the main graphs. Fruit was incubated in deionized water for quantifying the water influx. The incubation solution was then lyophilized and analyzed by HPLC.

The efflux rates of glucose, fructose, sorbitol, and sucrose all increased significantly as the water influx rate increased (Figure 4).

It is interesting, that in early stage III, the composition of the efflux from the ‘Burlat’ closely matched that of the juice of the ‘Burlat’ cherries. This close correspondence did not apply for the mature ‘Burlat’ nor in the ‘Regina’. For the mature ‘Burlat’ and in the ‘Regina’, the efflux was surprisingly rich in sorbitol, as compared to the composition of the expressed juice of fruit from the same batch. Furthermore, in ‘Regina’ the contribution of sorbitol increased from the time of early stage III to that of mature stage III (Table 1).

**Figure 5:**
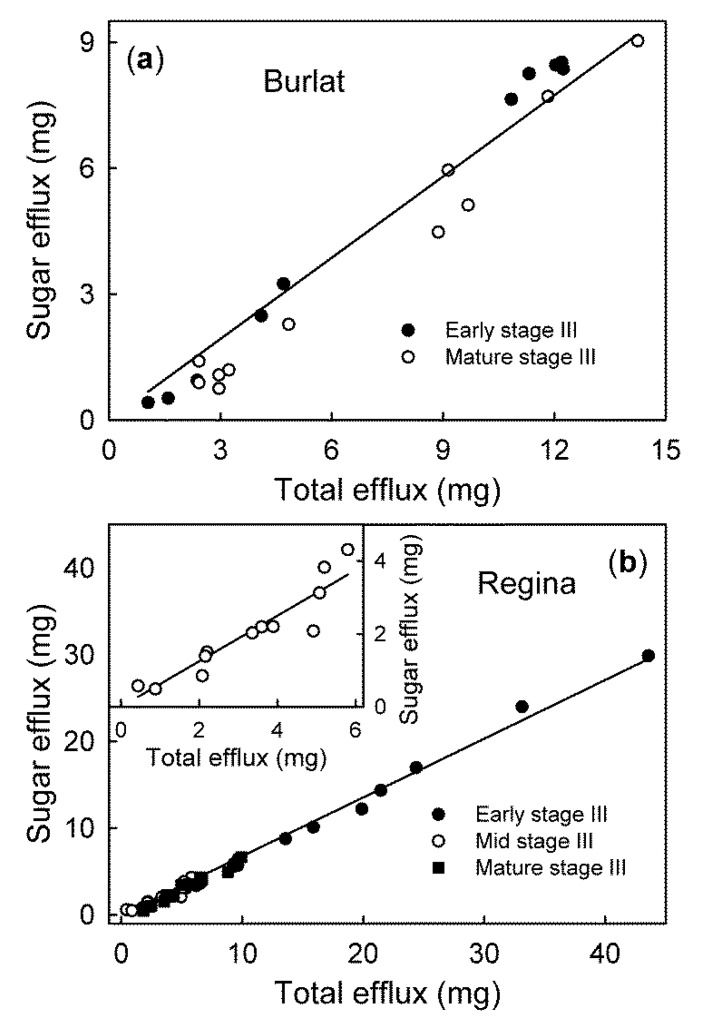
Relationship between the efflux of the sugars glucose, fructose, sorbitol, sucrose, and the total efflux of solutes in the ‘Burlat’ (**a**) and the ‘Regina’ sweet cherry fruit (**b**). These are the major osmolytes in sweet cherry that were quantified by HPLC.

For ‘Burlat’ and ‘Regina’, the sum of these carbohydrates accounted for 64% (r^2^ = 0.94 ***) and 68% (r^2^ = 0.99 ***), respectively, of the gravimetrically detectable efflux (Figure 5).

**Figure 6:**
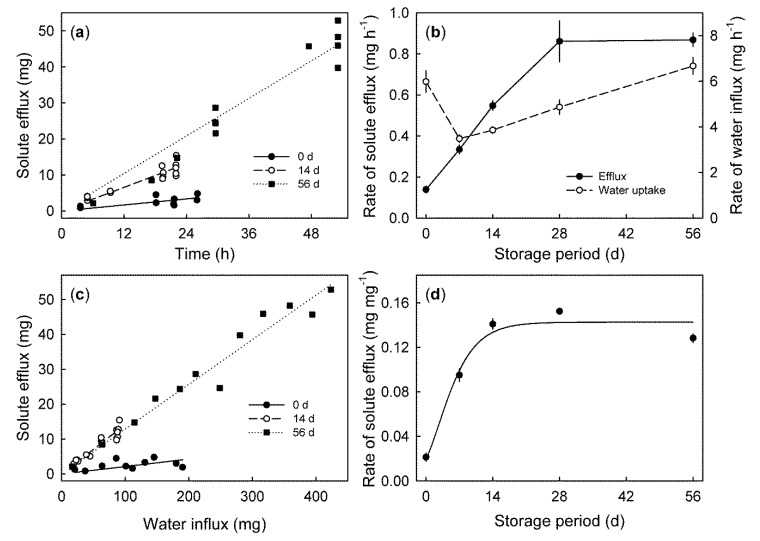
(**a**) Time course of the solute efflux of fruit that was stored for 0, 14, and 56 d at 2 °C and 90% relative humidity. (**b**) Rates of solute efflux and water influx, as affected by storage duration. (**c**) Relationship between solute efflux and water influx. (**d**) Efflux of solutes per unit water influx as affected by storage duration.

In fruit held in storage, the solute efflux also increased linearly with time. The rate of solute efflux was positively related to storage duration (Figure 6a). It increased during the first 28 days and thereafter remained constant for 56 days. The rate of water influx followed a different pattern. It decreased during the first 7 days and then increased up until 56 days (Figure 6b). The solute efflux increased linearly with the water influx. The relationship between the solute efflux and the water influx increased with increasing storage time (Figure 6c). Calculating the rate of the solute efflux per unit water influx revealed an increase up to a maximum, at 28 days, which remained constant thereafter (Figure 6d).

**Figure 7:**
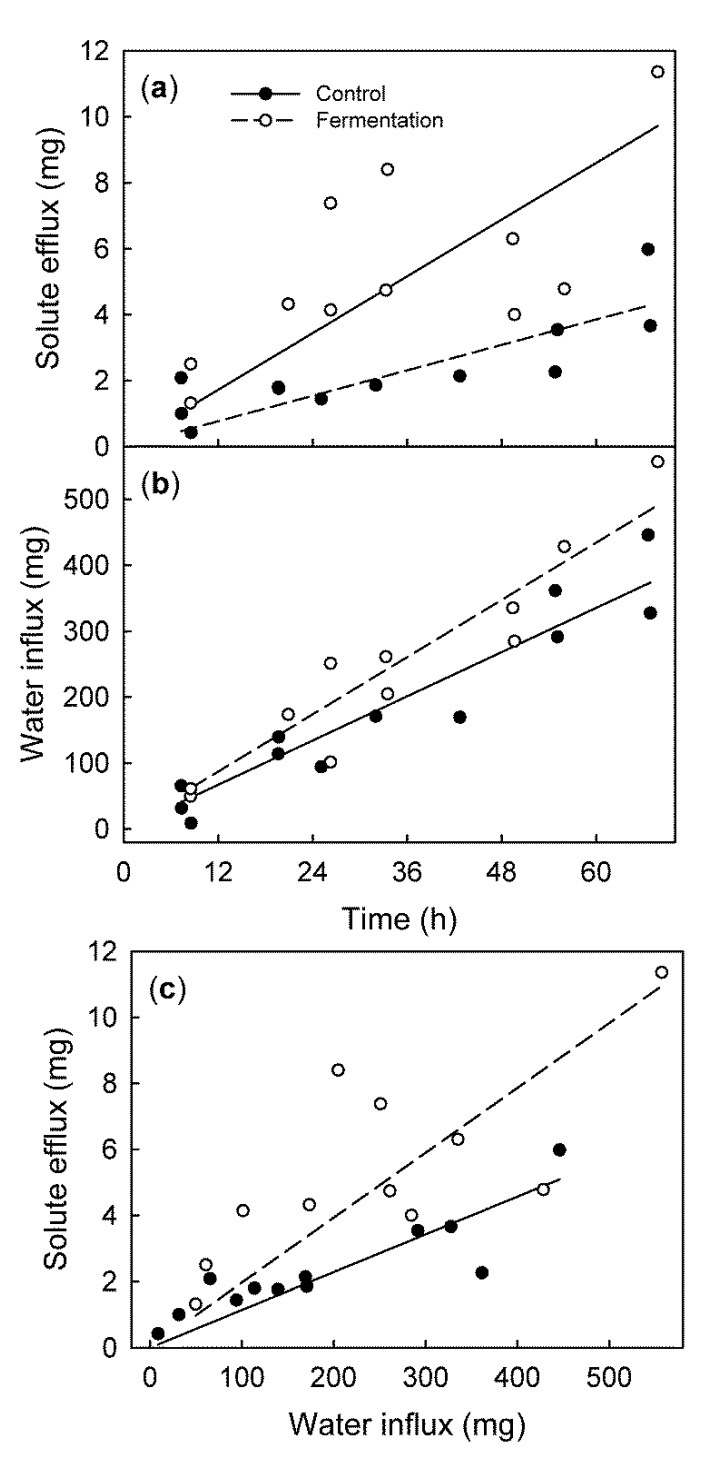
Time course of solute efflux from (**a**) and water influx into (**b**) the sweet cherry fruit. Fruit was induced to ferment by incubating it for 24 h in a sealed jar. The control fruit was allowed to respire. (**c**) Relationship between the efflux and water influx.

Fruit that began to ferment, as indicated by the significant production of ethanol, showed a higher rate of solute efflux, but there was no significant change in the rate of water influx (Figure 7a,b). Consequently, the solute efflux per unit water influx was higher for fermenting than for non-fermenting control fruit (Figure 7c).

## 3. Discussion

Our results demonstrated that during incubation in water, the sweet cherry fruit loses solutes (including anthocyanins) through their skins. The compositional balance of the major solutes lost—glucose, fructose, and sorbitol—largely reflects that in the expressed fruit juice. The rate of solute efflux increased preharvest, during fruit development and also postharvest during subsequent cool storage. The efflux was higher for the cracked and for the fermenting fruit than for the non-cracked and the non-fermenting control fruit.

### 3.1. Source of Solutes

The carbohydrates recovered from the incubation solution were glucose, fructose, and sorbitol and—at a low level—sucrose. Together, these four account for about 86.4% of the osmolytes in a sweet cherry fruit[6] and up to 69% of the gravimetrically measured efflux.

These solutes may be drawn from two different sources—the fruit symplast and the fruit apoplast. In early stage III ‘Burlat’, the composition of carbohydrates in the efflux matched that of the fruits’ expressed juice. Hence, these solutes represented the cell contents (the symplast) and not just the apoplastic solutes. In most intact (vital) tissues, the occurrence of these solutes was usually restricted to the vacuole. The surrounding tonoplast and plasma membrane served as effective, in-series penetration barriers [15]. Similarly, in intact tissues, the occurrence of the anthocyanins was usually restricted to the vacuole. The efflux of these solutes from the cytoplast into the cell-wall space and into the incubation solution implies that, both the tonoplast and the plasma membrane had suffered a significant loss of integrity. Such a loss is not unusual for mature, fleshy fruit, as has been shown for grape berries [11,12].

However, in mature ‘Burlat’ and in ‘Regina’, the sugar-alcohol sorbitol represented a surprisingly large fraction of the efflux; a fraction markedly larger than that predicted from the composition of the expressed juice from the fruit of the same batch. We do not know the reason for the preferential efflux of sorbitol. However, we do know that the sugars translocated in the phloem of the *Rosaceae* were characteristically high in sorbitol, along with the sucrose. Thus, the high rate of sorbitol efflux could be the result of a direct loss from the phloem, or a lack of loading into the adjacent phloem parenchyma cells, hence, the subsequent leakage of sorbitol through the skin from the fruit apoplast. To our knowledge, there is no published data on the composition of the phloem sap sugars in sweet cherry. In peach (also *Rosaceae*), the leaves were characteristically high in sorbitol, with a sorbitol:sucrose ratio ranging from 3.2 to 7.1 [16].

The negligibly low sucrose concentration in the solute efflux may be a result of (1) sucrose (MW 342) being a larger molecule than sorbitol (MW 182) or glucose (MW 180) or fructose (MW 180) and, hence, being less able to traverse the cuticle and/or (2) the immediate cleavage of any sucrose (a disaccharide) finding its way through the cuticle, into glucose and fructose (monosaccharides) through the enzyme invertase.

### 3.2. Mechanism of the Solute Efflux

Different mechanisms may account for the release of solutes from the vacuole to the cytoplast, and from there to the apoplast. These include (a) the bursting of cells due to excessive water influx and (b) programmed cell death associated with ripening and senescence. Both processes will result in the loss of membrane integrity.

(a) When a fruit is incubated in deionized water, water influx occurs through the cuticle and also through any cuticular microcracks. Here, the microcracks represent the sites of preferential influx, because the water can now bypass the cuticle, whose barrier function has been breached [17]. Following entry into the fruit, the influx of water is partitioned into the parenchyma cells of the flesh, just under the skin. Compared with the skin cells, the parenchyma cells are (1) larger, (2) have thinner walls, and (3) contain higher concentrations of osmotically-active solutes. The epidermal cells are smaller, thicker-walled, and their content is less concentrated [18]. For all three reasons, the parenchyma cells are the first to burst. As they burst, they release solutes and anthocyanins into the apoplast [19]. These solutes also include organic acids (malate) that increase the permeability of the membranes of the neighboring cells and also weaken their cell walls [20]. This process initiates a chain reaction that propagates through the flesh and into the skin like a ‘zipper’ in clothing. The chain reaction soon leads to a macroscopic crack [3,21]. As calcium is involved in the maintenance of membrane permeability and in the cross-linking of cell walls [22,23,24,25], calcium may play a role in this process. The stability of cell walls is positively correlated with its Ca-content [25,26]. Thus, cell walls with higher Ca-contents are less likely to crack than cell walls with lower Ca-contents.
(b) The second process causing solutes to be released from the cells into the apoplast is the last stage of fruit development—ripening and senescence. In general, senescence is accompanied by increases in membrane permeability and, thus, increases in leakage of all cytoplasmic solutes, including ions [27]. In grape berries, experimental evidence indicates a general breakdown of compartmentation [11]. This is likely also to occur in mature sweet cherry, where the regions of the flesh close to the pit appear to be entirely degenerated (Winkler and Knoche, unpublished data). Loss of membrane semipermeability quickly results in cell death. The production of reactive oxygen species (ROS) also causes cell death [28]. In sweet cherry, ROS result from increased activities during the storage of the enzymes superoxide dismutase, ascorbate peroxidase, lipoxygenase, guaiacol peroxidase, and polyphenol oxidase. Meanwhile, the activity of catalase as a part of the radical scavenging capacity in sweet cherry decreases. Additionally, the content of malondialdehyde as an indicator of membrane permeability increased [29]. All these processes are likely to contribute to increases in solute efflux during fruit storage.

### 3.3. Pathways for Efflux

The efflux rates of glucose, fructose, sorbitol, and sucrose were closely correlated with the rate of water influx, suggesting identical pathways for water influx and solute efflux. In principle, two pathways may be considered. First, polar pathways that are present in the sweet cherry [30], which result from the orientation of polar functional groups in the hydrated cuticular membrane (CM) [31]. They bypass the lipophilic CM and allow viscous flow, and diffusion of polar solutes and water in an aqueous continuum, across the CM [30,31,32]. Second, microcracks in the CM that form upon exposure of a strained CM to water [17]. The relative contribution of microcracks to the movement of water and solutes across the skin is unknown, but is expected to be highly variable.

### 3.4. Practical Implications

Our results have two implications. First, from a research point of view, we note that quantifying water influx to a fruit by incubating it in water (or in an aqueous solution) and recording changes in fruit mass, is considered both, a simple and a precise technique. It has been used in many studies with sweet cherry and also with other fruit species. However, we note that, based on this study’s findings, the mass changes inferred from periodic weighings of such a fruit will represent the result of both, a water influx and a solute efflux, and not solely that of a water influx. The procedural question, thus, arises as to how large are the errors involved in ignoring the mass rate of solute efflux? Our experiments demonstrate that both, the rate of water influx and also that of the solute efflux, depend on the stage of fruit development, the cultivar, and the time held in storage. Therefore, the answer is not entirely straightforward. Nevertheless, the gross change in fruit mass for a mature fresh fruit (no storage) lay in the range of 5.6 ± 0.3 to 10.3 ± 0.9 mg h^−1^ (a positive amount); however, of this, between 0.06 ± 0.01 and 0.66 ± 0.10 mg h^−1^ was to be attributed to a solute efflux (a negative amount). If we allow for the solute efflux, we obtain a slightly higher rate of water influx, in the range 5.7 ± 0.3 to 11.0 ± 0.9 mg h^−1^. Expressed another way, we can say the solute efflux lowered the inferred water influx rate by between 1.1 and 6.4%. It is fair to say that, under most circumstances, a systematic error of this magnitude may be considered negligible. The magnitude of this error for an unknown batch of fruit, or for a different fruit cultivar or a different fruit species, etc., would require assessment before it could reasonably be considered negligible. A straightforward and robust procedure for this would be to determine the dry mass of the lyophilized incubation solution.

Second, from a more commercial point of view, it would seem inevitable that solute efflux will also occur naturally when a fruit is wetted in the field by rain, dew, etc. As the fruit dries, the major fruit osmolytes that traversed the cuticle (for a sweet cherry, these are glucose, fructose and sorbitol) will become concentrated on the fruit surface. This will provide a perfect culture medium for the growth of a range of epiphytic microorganisms, including symbionts, such as *Saccharomyces* spp. and pathogens, such as *Monilinia* spp. It would be interesting to record the population densities of such microorganisms on the surfaces of fruit, as a function of the amount of immediately-elutable sugar substrates and to record such population densities in fruit exposed to different environmental conditions, with respect to rainfall, dew, and continued surface dryness. For example, one might expect a fruit exposed to dew to have a larger quantity of surface-elutable substrates and a higher microorganism population compared with one that was exposed either to rain (which would have washed off some of the surface sugars) or to surface water (e.g., where there were no surface sugars).

## 4. Materials and Methods

### 4.1. Plant Material

Sweet cherry fruit of the cultivars Burlat and Regina were sampled from the trees grafted on ‘Gisela 5′ rootstocks (*P. cerasus L. x P. canescens* Bois). The trees (planted in 2010) were trained as a slender spindle and grown under a rain shelter at the Horticultural Research Station of the Leibniz University in Ruthe, Germany (lat. 52°14′ N, long. 9°49′ E) in the 2018 and 2019 growing seasons (temperature profiles are displayed in Figure S2). All fruit were cultivated according to the current European regulations for integrated fruit production. Irrigation was supplied as needed, using microsprinklers, underneath the tree. Only uniform fruit free from visual defects were selected. The pedicels were cut flush with the receptacle. The pedicel cavity, pedicel end, and the pedicel/fruit junction were sealed using a fast curing silicone rubber (Dow Corning SE 9186; Dow Corning Corp., Midland, MI, USA). The silicone was allowed to cure for about 1 h. This treatment limited the water influx and the solute efflux to the fruit surface [33]. All experiments were carried out in a temperature-controlled laboratory at 22 °C.

### 4.2. Quantifying Efflux

Fruits were weighed and incubated individually in beakers containing 30 mL of deionized water. At regular intervals, some of the fruits were removed, carefully blotted using tissue paper, and re-weighed. At these times, the fruits were carefully inspected for cracks. Unless specified otherwise, all analyses were restricted to data for the non-cracked fruit. The incubation solutions from the non-cracked fruit were promptly frozen (−20 °C), pending further analysis. This procedure was continued until all fruits had cracked or had begun to rot. The incubation solutions were lyophilized, and the masses of the residues were determined gravimetrically (ME235P; Sartorius, Goettingen, Germany). Subsequently, the residues were taken up in 2.5 mL of deionized water and the soluble solids content was determined using a refractometer (DR6200-T; A. Kruess Optronic, Hamburg, Germany). The total anthocyanin contents were quantified by determining absorption at 520 nm, using a photometer (Specord 210; Analytik Jena, Jena, Germany) [34]. As anthocyanin absorption depends on pH, malic acid was added to a final concentration of 70 mM before the absorption rate was measured.

### 4.3. Experiments

The effluxes of solutes from cracked and from non-cracked fruit were established in ‘Regina’. The number of replicates was 12.

Developmental time courses were established in ‘Burlat’ at 27, 35, 41, 49, and 57 DAFB and in ‘Regina’ at 25, 46, 55, 63, and 77 DAFB. These five timings represent the fruit development stage II, the transition from stage II/III (as indicated by the onset of color change from green to straw yellow), early stage III, mid stage III, and maturity, respectively [13,14]. The osmotic potentials of the fruit’s expressed juice were measured using water vapor pressure osmometry (VAPRO^®^ 5520 and 5600; Wescor, Logan, UT, USA). The minimum number of replicates was 10.

The effect of storage duration on solute efflux was studied in ‘Regina’. Harvested fruit were held for 0, 7, 14, 28, and 56 days, at 2 °C and 90% relative humidity. The minimum number of replicates was 10.

The effect of fermentation on solute efflux was studied in ‘Regina’. Fruits were incubated at an ambient temperature, for 24 h in a gas-tight jar (76 fruits in a 1.5 l jar). After this period, the ethanol concentration in the headspace was determined through gas chromatography (GC 6850; Agilent, Santa Clara, CA, USA) [35]. The operating conditions were: temperatures of the inlet and of the flame ionization detector 200 °C and 300 °C, respectively. Gas flow rates 35 mL min^−1^ for H_2_, 400 mL min^−1^ for air, and 3 mL min^−1^ for He as carrier gas. The minimum number of replicates was 11.

### 4.4. HPLC Analysis of Carbohydrates

For the ‘Burlat’ (early and mature stage III) and the ‘Regina’ fruit (early, mid, and mature stage III), the amounts of glucose, fructose, sorbitol, and sucrose in the leachate were quantified according to the protocol used by Wang et al. [36]. A 20 µL aliquot of the solution used for refractometry was injected onto a HC-75 cation exchange column (Ca^2+^ form, 9 µm, 7.8 × 305 mm; Hamilton Company, Reno, NV, USA) of an HPLC system (LC-CaDI 22-14, HPLC compact pump 2250; Bischoff, Leonberg, Germany). The system was equipped with a refractive index detector (RI detector 8020; Bischoff) and a column heater set at 80 °C (Variotherm 880; Bischoff). Separation was carried out isocratically using deionized water as the mobile phase (flow rate 0.4 mL min^−1^). The number of replicates ranged from 11 to 17 for the leachates and was three for the fruit juices.

### 4.5. Data Analyses

Data reported in the figures are observations on individual fruits. The exceptions are in Figure 2a, Figure 3a and Figure 4c,d, where means ± SE are given. When the error bars were not visible in the latter figures, they were smaller than the plotted symbols. Comparisons of the treatment means were carried out using Students multiple *t*-test and the Bonferroni adjustment of *p*-values (*p* ≤ 0.05, R version 3.6.1; R Foundation for Statistical Computing, Vienna, Austria). Regression analyses were carried out using SigmaPlot (version 12.5; Systat Software Inc., San Jose, CA, USA) and R (R version 3.6.1; R Foundation for Statistical Computing). Significance of the coefficients of determination (r^2^) at *p* ≤ 0.05, 0.01, and 0.001 were indicated by *, **, and ***, respectively.

## Figures and Tables

**Table 1:** Carbohydrate compositions of juice extracted from sweet cherry fruit and of the efflux from fruit incubated in deionized water. These carbohydrates account for 86.4% of the osmolarity of the fruits’ juice. The number of biological replicates was three for the juices, and 11 to 17 for the solute efflux samples.

			Relative Content of the Sugars (%)			
Cultivar	Developmental Stage	Sample	Glucose	Fructose	Sorbitol	Sucrose
Burlat	early stage III	Fruit juice	47.4	39.7	8.3	4.7
		Solute efflux	35.7 **	33.9	20.6 *	9.8
	mature stage III	Fruit juice	47.9	40.4	9.3	2.4
		Solute efflux	25.0 ***	28.7 *	39.0 ***	7.3 *
Regina	early stage III	Fruit juice	50.0	35.8	11.4	2.8
		Solute efflux	24.6 ***	21.9 ***	48.9 ***	4.5
	mid stage III	Fruit juice	46.1	38.0	13.6	2.3
		Solute efflux	8.3 ***	10.6 ***	73.9 ***	7.3 ***
	mature stage III	Fruit juice	46.8	37.1	14.3	1.8
		Solute efflux	12.4 ***	11.0 ***	67.3 ***	9.2 **

Significant differences within columns, developmental stages and cultivars at *p* ≤ 0.05, 0.01, and 0.001 indicated by *, **, and ***, respectively, Students multiple *t*-tests.

## Supplementary Materials

The following are available online at https://www.mdpi.com/2223-7747/9/4/440/s1. Figure S1: Relationship between the soluble solid content of the efflux, as determined by a refractometer and its dry mass. Figure S2: Temperature profiles in the growing seasons 2018 (**a**) and 2019 (**b**).
